# New hydrate formation methods in a liquid-gas medium

**DOI:** 10.1038/srep40809

**Published:** 2017-01-18

**Authors:** A. A. Chernov, A. A. Pil’nik, D. S. Elistratov, I. V. Mezentsev, A. V. Meleshkin, M. V. Bartashevich, M. G. Vlasenko

**Affiliations:** 1 Kutateladze Institute of Thermophysics, Siberian Branch of the Russian Academy of Sciences, Novosibirsk 630090, Russia; 2 Novosibirsk State University, Novosibirsk 630090, Russia

## Abstract

Conceptually new methods of hydrate formation are proposed. The first one is based on the shock wave impact on a water-bubble medium. It is shown that the hydrate formation rate in this process is typically very high. A gas hydrate of carbon dioxide was produced. The process was experimentally studied using various initial conditions, as well as different external action magnitudes. The obtained experimental data are in good agreement with the proposed model. Other methods are based on the process of boiling liquefied gas in an enclosed volume of water (explosive boiling of a hydrating agent and the organization of cyclic boiling-condensation process). The key features of the methods are the high hydrate formation rate combined with a comparatively low power consumption leading to a great expected efficiency of the technologies based on them. The set of experiments was carried out. Gas hydrates of refrigerant R134a, carbon dioxide and propane were produced. The investigation of decomposition of a generated gas hydrate sample was made. The criteria of intensification of the hydrate formation process are formulated.

Over the recent years the interest in gas-hydrates has grown all over the world^1^,^2^,^3^. Previously, the majority of studies were aimed at finding the methods of prevention of hydrate formation and avoiding solid phase accumulation in systems of underground and overground equipment at oil and gas deposits^4^,^5^,^6^. But now the emphasis is shifted towards the perspective of using hydrates and the hydrate formation process in practice. Thus, for example, one economically sound method of gas transport in the absence of gas pipeline involves converting gas to gas-hydrate, transporting it in a solid state under static pressure and low temperature (although, recently increasingly greater attention has been paid to the transportation under non-equilibrium conditions and atmospheric pressure)^7^,^8^,^9^. Such transportation method is the most profitable for small oil-gas fields and the collateral effect can be achieved by using both the gas and clean water remained after gas-hydrate decomposition. Another use of hydrate technologies is the gas storage (in the gas-hydrate state) near large consumers^10^,^11^,^12^. Artificial hydrate formation processes can also be used outside the oil and gas industry for sea water desalination, gases separation, fog elimination, heat accumulation, creation of efficient refrigeration cycles and others.

Gas-hydrate technologies can also help solve global ecological problems^13^,^14^,^15^,^16^,^17^. The major ecological problem is the climatic change, which is connected with the increase in the concentration of greenhouse gases including carbon dioxide. Obviously, the release rate of carbon dioxide will increase with the growth of industrial production. One promising, for the large-scale use, method of gases utilization involves gas conversion into the gas-hydrate state and storing it at the ocean bottom under low temperature and high pressure.

Obviously, the main factor ensuring the financial viability of such technologies is the rate of hydrate formation. As a result, we set the objective to develop a fast and cost-effective method of hydrate formation. The currently existing technologies of hydrate formation are based on: intensive mixing of gas-saturated water^18^, fine water jet dispersion in gas atmosphere^19^, vibratory and supersonic influence on a bubble medium and so on. However, the majority of above-mentioned methods can be characterized by a low hydrate formation rate and, as a result, low efficiency of plants that consume them.

All the research of gas hydrates can be divided into two parts: fundamental and applied. The most important of them are the studies of a gas-hydrate structure, its physicochemical, thermophysical, mechanical and other properties, the general conditions needed for their formation and their growth mechanisms^20^,^21^,^22^,^23^,^24^,^25^,^26^,^27^,^28^. Great attention is paid to the methods of studying both natural and man-made gas hydrates^29^,^30^,^31^,^32^,^33^,^34^,^35^,^36^,^37^. A lot of contributions are dedicated to the enhancement of the hydrate formation process. A great deal of articles are devoted to experimental and mathematical modeling of gas hydrates formation and decomposition processes^38^,^39^,^40^,^41^. There are many patents for hydrate formation methods and big corporations, such as Mitsui, Toyota, Chevron and others are their holders.

Comparatively recently, a new hydrate formation method has been proposed. It is based on the shock wave impact on a bubble medium^13^,^14^,^15^,^42^. It was shown that the main mechanism responsible for the high rate of hydrate formation is related to the fragmentation of bubbles in the shock wave during their active mixing. It leads to intensification of the processes of heat and mass transfer at the interphase and, as a result, to the intensification of the hydrate formation process.

In this work we propose new hydrate formation methods based on the mechanical or/and thermal influence on a bubble medium.

## Some information about gas hydrates

Gas hydrate is a solid crystalline compound that forms, under certain temperature and pressure conditions, from water (liquid water, ice or water vapor) and a low-molecular gas^5^,^6^. Gas hydrates are categorized as the so-called clathrate compounds or compounds where gas molecules (“guest”) are trapped in the molecular cavities of an ice-like molecular structure (“host”) formed by water molecules via hydrogen bonds. Gas molecules (“guests”) are bonded with the structure (“host”) only by Van der Waals forces without forming chemical bonds. The general formula of a gas hydrate is as follows: M*n*H_2_O, where M means the guest molecule; *n* — number of water molecules per one guest molecule. Number *n* is a variable and depends on the type of gas, pressure and temperature.

X-ray, NMR spectroscopy, neutron diffractometry and crystal-chemical modeling show that the cavities of the structure usually are 12– (“small” cavities, referenced as D and D′), 14–, 16– and 20–hedrons (“large” cavities, referenced as T, T′, P, H and E), slightly deformed related to the ideal form. The vertices of the structure are oxygen atoms of and the edges are hydrogen bonds. The most effective one, in terms of energy, is the D–cavity (or pentagonal dodecahedron). Combining with each other the cavities form a compact structure of different types. According to the accepted classification, they are labeled “s” and “H” (cubic and hexagonal structures). Types sI and sII are the predominant in nature and other structures seldom form and are often metastable. The unit cell of sI consists of 46 water molecules and they form 2 small cages (D) and 6 large ones (T). The unit cell of sII consists of 136 water molecules and they form 16 small cages (D) and 8 large ones (T). The studies of gas hydrates phase diagram, especially under high pressure, show that several hydrate structures can appear in one system.

One of the unique properties of gas hydrates is the ability of trapping 70 to 300 gas volumes for one water volume. For example, 1 m^3^ of methane hydrate can carry 160 m^3^ of gas and the volume occupied by gas in a gas hydrate is less than 20%. The density of gas hydrate is typically less than the density of water and ice (for methane hydrate it is around 900 kg/m^3^). After the increase in temperature and the decrease in pressure the hydrate decomposes into gas and water absorbing great amount of heat in the process.

## Hydrate formation caused by the shock wave impact in a water-bubble medium

### Experimental setup

**Figure 1:**
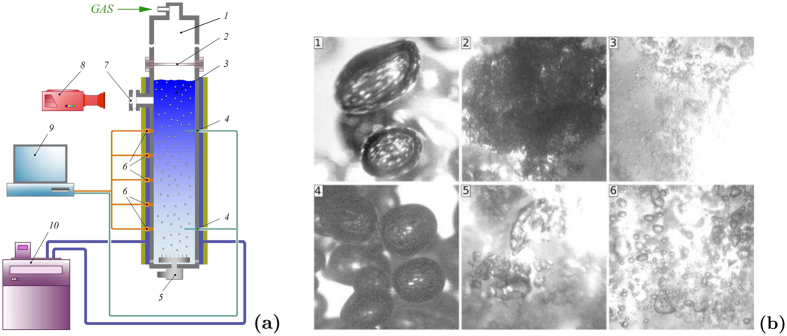
(**a**) Scheme of experimental setup: 1–high pressure chamber; 2–diaphragm; 3–working section; 4–thermocouples; 5–gas bubble generator; 6–pressure piezosensors, strain sensor, conductivity sensor; 7–optic window; 8–photo camera; 9–ADC & PC; 10 – cryostat. (**b**) Photo of carbon dioxide bubbles behind the front of shock wave at different moments: *P*_0_ = 0.5 MPa, Δ*P*/*P*_0_ = 1.72, *t* = 1 ms (1), 2.7 ms (2), 7.9 ms (3); *P*_0_ = 1.3 MPa, Δ*P*/*P*_0_ = 0.67, *t* = 1 ms (4), 4 ms (5), 9.8 ms (6).

Experiments were performed at the “shock tube” setup, Fig. 1(a). The experimental setup consists of the vertical steel pipe (internal diameter–53 mm), that can endure a high pressure (up to 10 MPa). The bottom of the working section is sealed from the atmosphere by the bulkhead. On the top it is sealed from the high pressure chamber by the membrane. The length of the working section is 1.5 m. In the experiments, the working section was filled with bubble bearing water (with hydrating gas bubbles) with a high volumetric gas content (up to 60%). Carbon dioxide was used as a hydrating gas. Some experiments were carried out in the presence of N-octanol (surfactant) in the medium. The average radius of bubbles in the experiment was determined by a photographic recording of the medium through the optic window with a high resolution camera (initial radius was around 2–3 mm). The volumetric gas content was measured with the conductivity probe (with rapid response) placed at the top of the working section. All experiments were carried out under the constant temperature *T*_0_ 1°C reached by pumping a cooling liquid between the wall of a working section and the jacket covered with an insulator. Thermocouples were built in the wall of the working section for temperature control. The initial pressure *P*_0_ in the medium was held higher than the atmospheric pressure, but lower than the pressure of hydrate formation (for the given temperature) and ranged from 0.5 MPa to 1.3 MPa. It was reached by the use of the electromagnetic valve controlled by the tension gauge placed in the working section. The membrane rupture (separating the working section from the high pressure chamber) generated shock waves of different magnitudes. The shock wave intensity was held at a level where the hydrate formation was possible in the medium behind the wave front.

### Method description

#### Results

Let us consider a liquid with gas bubbles where a 1D shock wave propagates. The following processes occur behind the front of a shock wave: isothermal compression of bubbles, their involvement in the movement related to the liquid and the bubbles fragmentation (due to Kelvin–Helmholtz instability) in the case of a shock wave of a high magnitude. The fragmented bubbles form the so-called gas-liquid clusters with a developed phase contact area (Fig. 1(b)). The way shock waves are generated is chosen so that, behind the shock wave, the medium will fall into the area where the hydrate formation from the gas in gas bubbles is possible. The hydrate shell growing on the surface of moving bubbles has a porous structure (can be presented as overlapping flakes of the hydrate) and, apparently, it does not create any substantial resistance to the gas flowing through it towards the liquid. Thus, the growth rate of hydrate mass in this process is limited not only by diffusion (as in the case of hydrate forming in gas pipes), but by a convective heat transfer from the interphase. Let us note that if the gas is highly soluble in water, the dissolution process can significantly hamper the hydrate formation process.

Using the suggested method, a series of experiments was carried out using different initial conditions (initial pressure, surfactant concentration, etc.) and different intensities of shock-wave exposure (magnitude of generated shock waves Δ*P* ranged from 2 MPa to 4 MPa). Carbon dioxide was used as a hydrating gas. It is highly soluble in water and, as mentioned before, the solubility hinders the hydrate formation process. The experiments show that the bubble size in a cluster (after the fragmentation of initially existing bubbles) decreases with the increase of shock wave magnitude from 150 *μ*m to 25 *μ*m, and their volumetric concentration in cluster *φ*_*cl*_ increases by 50% to 25%, which makes sense.

The mathematical model of the considered process can be found in refs 13 and 14. The model includes the equation for the mass of gas in bubbles, which determines a change in the gas phase volume concentration in liquid. The mass and energy balance equations were written for the interphase. To describe the processes of bubbles dynamics and heat and mass transfer, the well-known solutions were used. Heating a liquid in a cluster due to the phase transition was taken into account in the model using the energy balance equation. The interaction between the diffusion layers around the bubbles in the cluster was neglected.

**Figure 2:**
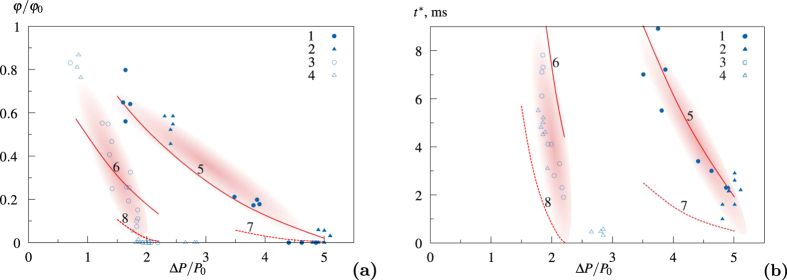
Dependences of dimensionless volumetric gas content *φ*/*φ*_0_ behind the shock wave front after 9 ms (**a**) and the time of hydrate formation t* (**b**) on dimensionless wave amplitude Δ*P*/*P*_0_: 1, 3–experimental data (without surfactant); 2, 4–experimental data (with surfactant); 5, 6–calculated dependences for *φ*_*cl*_ = 50%; 7, 8–calculation dependences for *φ*_*cl*_ = 25%; *P*_0_ = 0.5 MPa (1, 2, 5, 7), *P*_0_ = 1.3 MPa (3, 4, 6, 8).

The experimental results are shown in Fig. 2. Figure 2(a) represents the dimensionless volumetric gas concentration in liquid *φ*/*φ*_0_ at the moment *t* = 9 ms after the passing of shock wave through the pipe cross section, and Fig. 2(b) — the characteristic time of hydrate formation (time, when the relative volumetric gas content becomes equal to the value 0.2) on dimensionless wave amplitude Δ*P*/*P*_0_ = . A comparison with the results of numerical calculations using the above-mentioned model was made. It can be seen in Fig. 2 that the results are in good agreement. The relatively big scatter of experimental data is caused by a fairly wide size distribution of the initial bubbles, as well as by the variety of shapes and sizes of the formed gas-liquid clusters (see Fig. 1(b)). The analysis of the carried out experiments allows making certain brief conclusions. It can be seen in Fig. 2(a) that, with the increase in Δ*P*, the volumetric concentration of gas phase *φ*/*φ*_0_ in 9 ms from the start of the process decreases. This means that, for the shock waves of higher magnitude, the rate of hydrate formation is higher. The same conclusion can be drawn by the analysis of the data shown in Fig. 2(b). It can be seen that the time when volumetric gas concentration *φ*/*φ*_0_ reaches 0.2 decreases with the increase of Δ*P*. It can be explained by the fact that a more developed phase contact area can be formed behind the stronger shock wave in a gas-liquid medium. Also the bubbles behind the front of the shock wave have higher velocities. Thus, the heat transfer from the interphase is more intensive. And the heat transfer largely determines the growth rate of the hydrate mass. Also, the additional effect is provided by the fact that, behind the shock wave of a higher magnitude, the medium falls in the area of a higher decompression related to the hydrate formation curve (which increases the hydrate mass growth rate). The enhancement of this effect can be achieved by making the initial pressure close to the equilibrium pressure for a given temperature (in the described experiments it is 1.3 MPa). Against all expectations the surfractant does not have the significant influence on the hydrate formation process (it was expected that the decrease in the surface tension caused by the surfractant would lead to the formation of a more developed phase contact area). It can be seen from the experimental results (Fig. 2). The experimental points for experiments with and without a surfactant lie almost in the same region. Let us note that, during the experiments, the maximum amount of the gas converted to the gas hydrate was about 60% (for *P*_0_ = 1.3 MPa and Δ*P*/*P*_0_ ~ 2). The remaining gas dissolved in water.

## Hydrate formation at the explosive boiling of a liquefied gas in a water volume

### Experimental setup

**Figure 3:**
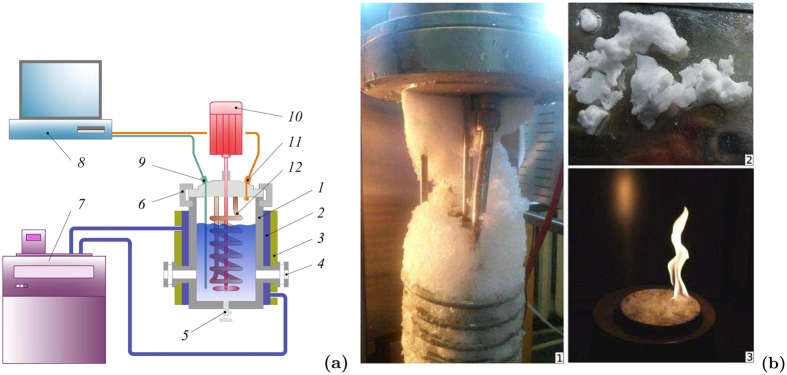
(**a**) Scheme of experimental setup: 1–vessel; 2–cooling jacket; 3–insulator; 4–optical window; 5–bottom valve; 6–lid; 7–cryostat; 8–ADC & PC; 9–tube with a thermocouple; 10–mixer drive; 11–pressure gauge; 12–cooling coil. (**b**) The hydrates of different gases produced in the experiments: 1–refrigerant R134a; 2–carbon dioxide; 3–propane.

All experiments were conducted using the autoclave (Fig. 3(a)). The working section is the reactor with a cylindrical chamber of 100 mm in diameter and the height of 300 mm made of stainless steel that can endure the pressure of up to 25 MPa. The working section is thermostated by cooling jacket. It consists of a coil pipe for an additional heating or cooling of the liquid. The temperature needed for the hydrate formation is provided by the low-temperature cryostat LOIP FT-316-40. The chamber is equipped with viewing windows for monitoring the processes taking place inside it. We can mix the liquid using a built-in mixer. The inside of the chamber can be accessed through the lid on top. The lid tightly closed using two semiring cramps and a couple of valves. The safety relief valve, cooling coil, pressure gauge, pressure sensor and thermocouple are attached to the lid. The needle valve for draining the liquid after the experiment is placed at the bottom of the chamber. The control unit on the nearby rack is used to operate the autoclave. Different gas hydrates produced in the experiments are presented in Fig. 3(b).

### Method description

#### Results

The main principle of the proposed method is as follows. Water in the chamber is cooled down to a temperature close to 0 °C; later the gas is supplied into the chamber. The pressure in the gas container from which the gas is supplied is significantly larger than the pressure in the chamber and it is kept at room temperature. The pressure in the chamber rises, the gas cools down and condenses to the liquid state. As a result, the liquid either collects at the bottom of the chamber or stays on the surface of the water (depending on the liquefied gas density). Later, we create a decompression by venting the gas (with the rate of about 1 liter/s). As a result, the liquefied gas at the bottom of the chamber boils explosively and intensively mixes with water (additional mixing is needed if the liquid is on the surface of the water). This leads to the formation of a developed surface. In addition, gas bubbles become involved in the motion related to the surrounding liquid. It is worth noting that the liquid around the bubbles cools down significantly (due to the phase change). In other words, the medium falls into the domain of the existence of gas hydrate. It leads to the formation and growth of hydrate shells around the bubbles. The surface of the bubble always undergoes the external action, because of the active boiling. Due to this, the shell has a discontinuous nature (or peels off as flakes). Therefore, the rate of hydrate formation is not limited by diffusion and is driven by the heat transfer (which is considerable in this case). All these lead to a rapid growth of the hydrate mass throughout the whole volume. Let us note that the whole hydrate formation process lasts only a couple of seconds.

**Figure 4:**
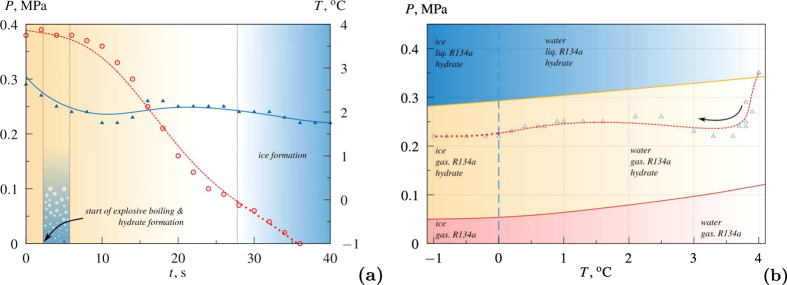
(**a**) Dependences of pressure *P* (triangles, solid line) and average temperature *T* (circles, dashed line) in the working section on time *t*. (**b**) The process in the *P* − *T* Diagram (triangles, dashed line); top solid line–boiling curve^43^; bottom solid line–hydrate formation equilibrium curve^44^.

The gas hydrates of refrigerant R134a, carbon dioxide and propane were produced in the experiments. Figure 4(a) shows the dependences of pressure and average temperature in the working section on time in the experiment of R134a hydrate formation. The ideal unit cell formula of the R134a hydrate is known to be 8R134a · 136 H_2_O, the hydrate crystal structure II. Thus, the gas mass fraction *k* in the pure hydrate is 25%. As we can see in Fig. 4(a), the venting of gas leads to a rapid pressure drop in the system. This is the cause of the explosive boiling of liquefied gas. Intensive boiling leads to a insignificant (transient) increase in pressure. Further, when the intensity of the boiling decreases, the pressure gradually drops to the ambient pressure. Also the average temperature of the medium decreases (due to phase transition and external cooling) and reaches sub-zero levels.

Figure 4(b) illustrates the process in the *P* − *T* Diagram. It shows that the process starts in the region where the refrigerant exists in the liquid state and proceeds to the region where it exists in the gaseous state. It is worth noting that, in both regions, the hydrate formation is possible. But the process of explosive boiling initiated by decompression significantly enhances the hydrate formation process (the rate of hydrate formation increases by several orders of magnitude).

**Figure 5:**
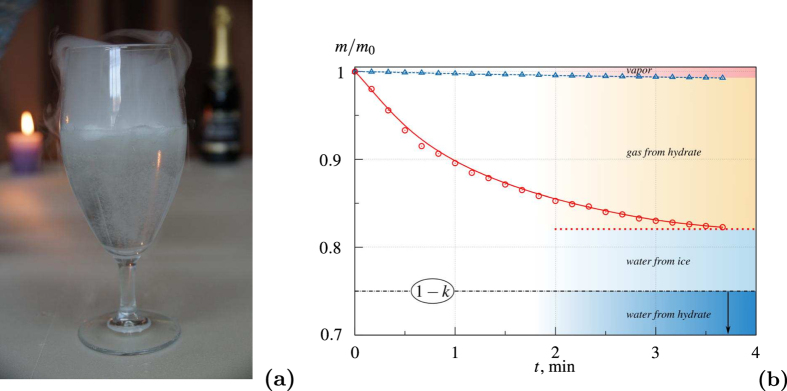
(**a**) Photo of hydrate decomposition in warm water. (**b**) Dynamics of degassing of hydrate sample produced in the experiment during the decomposition (solid line); dynamics of decomposition of the same amount of ice (dashed line): *m*–mass of the sample during decomposition; *m*_0_–initial mass of the sample; *k*–mass fraction of the gas in the hydrate.

The diffraction patterns confirm the presence of a substantial fraction of hydrate in the solid phase (which contains a gas hydrate and ice). The investigations carried out in the dynamics of samples degassing during the decomposition also show this fact. The method was as follows. The produced solid mass was placed in an open vessel with warm water and it starts to thaw releasing a copious amount of gas (Fig. 5(a)). The dynamics of degassing shows us the gas hydrate content in the sample and the amount of gas in it. The plot of such dependence is shown in Fig. 5(b) for the hydrate of R134a. The figure shows that the initial mass concentration of the gas in the sample was about 20%; so, about 70% of the sample were gas hydrate.

## Hydrate formation in the cyclic process of gas boiling-condensation in a water volume

### Experimental setup

**Figure 6:**
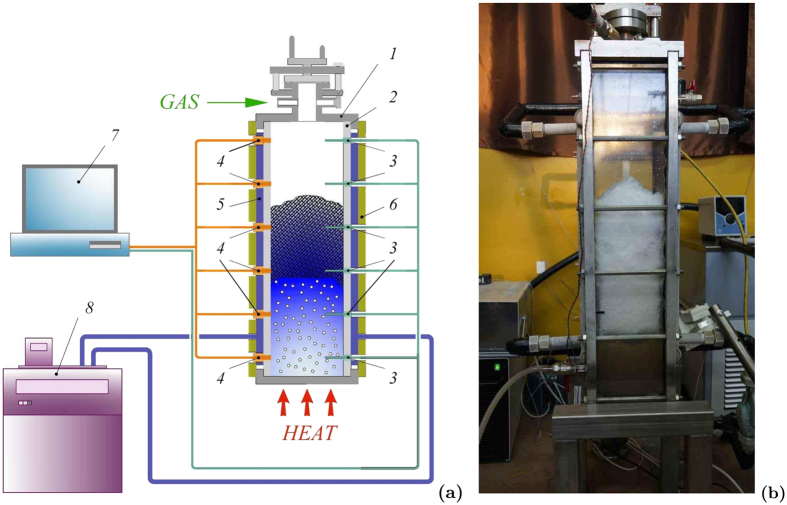
(**a**) Scheme of the experimental setup: 1–top lid; 2–side wall; 3–thermocouples; 4–pressure gauges; 5–cooling jacket; 6–insulator; 7–ADC & PC; 8–cryostat. (**b**) Gas hydrate of the refrigerant R134a produced during the experiment.

All experiments were made using the following experimental setup. The working area is a parallelepiped with the overall dimensions of 740×150 × 150 mm made of stainless steel with the wall thickness of 15 mm (Fig. 6(a)). Two viewing windows allow us to register the processes inside the chamber using the photo and video equipment. Detectors PD-100 and DTS204-RT100 were used for measuring the pressure and temperature. This experimental setup allows us to study the hydrodynamic and thermophysical processes under high pressure (pressure ranges from 0.1 to 10 MPa) and under low temperatures (down to −10 °C). The cooling is done through the side walls using a cooling jacket. The bottom of the chamber is heated to reach the intensive boiling of the liquefied gas. Refrigerant R134a was used as a gas for the hydrate formation. The hydrate of R134a produced in the experiment is presented in Fig. 6(b).

### Method description

#### Results

The main principle of the proposed method is as follows. The working section is half filled with water under excessive pressure *P*_0_ = (0.33 − 0.38) MPa. The water is cooled down to temperature *T*_0_ = (2 − 5)°C. After this the gaseous refrigerant is supplied into the chamber. The refrigerant rapidly condenses on cold walls and streams down to the bottom of the unit, and forms a liquid layer. It starts to boil at a high rate, because of the organized heating. The bubbles formed during boiling rise to the cool area (where hydrate formation is possible) and, as a result, the gas hydrate shell starts to form (shaped as flakes pervious to gas). The hydrate shell growth rate is defined by the heat dissipation into the surrounding liquid (because gas has always an access to water and the diffusion resistance can be disregarded). After rising up to the free surface of the liquid and collapsing, the bubbles leave their flakes of gas hydrate. The refrigerant remained from the hydrate formation in the first cycle of the process condenses on the walls of the working section, streams down to the bottom of the chamber and mixes with the already boiling layer.

**Figure 7:**
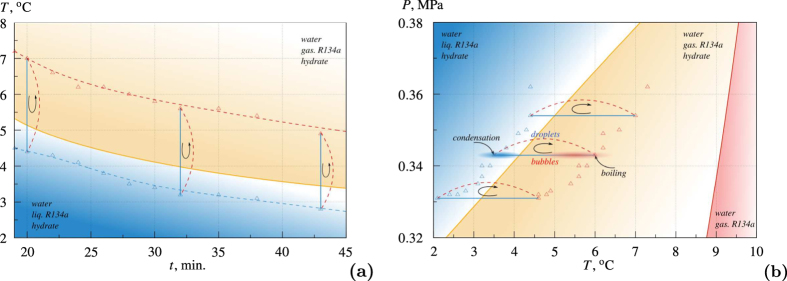
(**a**) Dependence of liquid temperature *T* in the working section near the heater (top dashed line) and near the free surface (bottom dashed line) on time *t*; solid line — boiling curve^43^. The boiling temperature changes with time because of the changing pressure. (**b**) The process in the *P* − *T* Diagram; left solid line–boiling curve^43^; right solid line–hydrate formation equilibrium curve^44^. The cyclic processes of R134a boiling-condensation at different times of the process are schematically presented in the figure.

Figure 7(a) shows the dependence of liquid temperature in the working section near the heater (in this region the temperature is higher than the boiling point during the entire process) and near the free surface (in this region the temperature is lower than the boiling point during the entire process) on time. Figure 7(b) illustrates the process in the *P* − *T* Diagram. The thermodynamic cycles, in which R134a is involved at different times of the process, are schematically presented in Fig. 7(a,b). Obviously, this cycles continue until all refrigerant transforms into the gas hydrate. Let us note that the rate of such process is restricted by the heat input at the bottom of the working section and heat sink at the walls of working section. We deal with the refrigeration cycle. The major part of the refrigerant turns into the gas hydrate in half an hour. The X-ray diffraction patterns confirm the presence of the hydrate substantial fraction in the samples (they consist of the gas hydrate and ice). Let us note that, despite the positive average temperature, some ice forms in the system. It is due to the fact that the temperature around the growing bubble during boiling can be significantly lower than the medium temperature.

## Conclusion

New hydrate formation methods were developed. All methods are based on intensive mechanical and/or thermal stresses in a gas-liquid medium. The first one is based on the shock wave impact on a water-bubble medium. This process includes the gas bubbles fragmentation at the front of the shock wave and their involvement in the movement related to the liquid. It enhances the heat transfer from the interphase and leads to a rapid hydrate shell formation around the bubble. The explosive boiling of liquefied gas in a closed water volume caused by a rapid decompression of the working section is implemented in the second method. This process involves the formation of a developed phase contact area, intensive mixing of liquid and gas phases and cooling of the medium caused by phase transition. All of the above-mentioned factors lead to a rapid formation and growth of a hydrate mass. The hydrate formation process lasts a couple of seconds. The cyclic process of hydrating gas boiling-condensation in the closed water volume is implemented in the third method. As in the previous method, the high rate of hydrate formation is caused by the formation of a developed phase contact area and by the presence of forced convection in the bubble-bearing liquid.

A set of experiments in the formation of carbon dioxide, refrigerant R134a and propane gas hydrates was carried out. The investigations in samples degassing dynamics during the decomposition process, as well as the analysis of diffraction patterns, show the presence of a substantial amount of gas hydrate in the solid phase.

We regard all the proposed methods as attractive alternatives to the already existing methods. They allow the formation of different gas hydrates over a short period of time consuming the modest amount of power. The whole process of the first method lasts only a couple of milliseconds but only the gas initially contained in the bubbles can be converted to a gas hydrate. The whole hydrate formation process in the second method lasts a couple of seconds. But, in this method, the substantial amount of gas in the chamber can be converted into the hydrate state. And the gas lost because of venting can be easily returned to the working volume in the next stage. The hydrate formation in the third method lasts half an hour. But, in this method, all the gas in the working volume eventually converts to the hydrate state.

Let us point out a set of conditions implemented in all the suggested methods which, in our opinion, lead to the intensification of a hydrate formation process. They can be briefly listed as follows. It is necessary to:

- create a developed interface surface;
- create the conditions when the medium is brought to the region of the most supercooling related to the hydrate phase equilibrium curve (to the region of the most metastable condition);
- create the conditions for a periodical fracture of growing on the interphase hydrate layer so that gas and water would always be in a direct contact and the hydrate formation would not be limited by diffusion only;
- ensure an intensive removal of heat from the interphase.

The ideas developed in the current research can be used for the creation of new energy-efficient and cost-effective commercial hydrate formation methods.

## Additional Information

**How to cite this article**: Chernov, A. A. *et al*. New hydrate formation methods in a liquid-gas medium. *Sci. Rep.* **7**, 40809; doi: 10.1038/srep40809 (2017).

**Publisher's note:** Springer Nature remains neutral with regard to jurisdictional claims in published maps and institutional affiliations.
